# Protective Effect of *Cleistocalyx nervosum* var. *paniala* Fruit Extract against Oxidative Renal Damage Caused by Cadmium

**DOI:** 10.3390/molecules21020133

**Published:** 2016-01-22

**Authors:** Warut Poontawee, Surapol Natakankitkul, Orawan Wongmekiat

**Affiliations:** 1 Department of Pharmaceutical Sciences, Faculty of Pharmacy, Chiang Mai University, Chiang Mai 50200, Thailand; warut.poontawee@hotmail.co.th (W.P.); surapolhsri@gmail.com (S.N.); 2 Department of Physiology, Faculty of Medicine, Chiang Mai University, Chiang Mai 50200, Thailand

**Keywords:** *Cleistocalyx nervosum* var. *paniala*, catechin, oxidative stress, antioxidant, cadmium, nephrotoxicity

## Abstract

Cadmium nephrotoxicity is a serious environmental health problem as it will eventually end up with end stage renal disease. The pathobiochemical mechanism of this toxic heavy metal is related to oxidative stress. This study investigated whether *Cleistocalyx nervosum* var. *paniala* fruit extract (CNFE) could protect the kidney against oxidative injury caused by cadmium. Initial analysis of the extract revealed antioxidant abilities and high levels of polyphenols, particularly catechin. Its potential renal benefits was further explored in rats treated with vehicle, CNFE, cadmium (2 mg/kg), and cadmium plus CNFE (0.5, 1, 2 g/kg) for four weeks. Oxidative renal injury was developed after cadmium exposure as evidenced by blood urea nitrogen and creatinine retention, glomerular filtration reduction, renal structural damage, together with increased nitric oxide and malondialdehyde, but decreased antioxidant thiols, superoxide dismutase, and catalase in renal tissues. Cadmium-induced nephrotoxicity was diminished in rats supplemented with CNFE, particularly at the doses of 1 and 2 g/kg. It is concluded that CNFE is able to protect against the progression of cadmium nephrotoxicity, mostly via its antioxidant power. The results also point towards a promising role for this naturally-occurring antioxidant to combat other human disorders elicited by disruption of redox homeostasis.

## 1. Introduction

Human illness caused by environmental pollutants and occupational-related toxic substances is a major public health problem currently evident in industrial societies, particularly in the developing countries. The Industrial Revolution led to a serious increase in the global metal pollution and increased production of heavy metals [1]. Cadmium (Cd) is one of the toxic heavy metals that is produced, about 13,000 tons/year, mainly from nickel-cadmium batteries, pigments, chemical stabilizers, metal coatings, and alloys [1]. It is also a toxic element of continuing concern because environmental levels have risen steadily due to continued worldwide anthropogenic utilization.

The kidney is well recognized as a critical target organ of cadmium toxicity. It is the site where 50% of cadmium is deposited, whatever the source and route of entry [2]. Due to its very long biological half-life of approximately 20–30 years [3], cadmium becomes a cumulative toxin throughout human life. Cadmium nephrotoxicity is really harmful as it causes renal failure and advances to end stage renal disease in the long run [2,4]. Although cadmium may induce renal injury through complex and multifactorial mechanisms, certain current evidence indicates the alteration in redox balance and induction of oxidative stress as the most important process [1,5,6,7].

*Cleistocalyx nervosum* var. *paniala* (*C. nervosum*), commonly known as Ma kiang, is found in Southeast Asia, especially in the northern part of Thailand [8]. It is an edible plant belonging to the Myrtaceae family. The fruit of *C. nervosum* is oval, no larger than 10–18 mm, having purple to black color when it is ripe, and is usually consumed in fresh or processed into juice, wine, tea, and jam. Recently, *C. nervosum* has been reported to possess antioxidant, antimutagenic, anticarcinogenic, and anti-aging properties [8,9,10,11]. However, most of the information related to these properties of *C. nervosum* was generated in isolated *in vitro* systems. Further investigation of the biological activities of *C. nervosum* in a particular condition using an *in vivo* model is needed.

Increasing interest in the use of phytochemicals on human health and disease prevention led us to explore the possibility of *C.nervosum* as a preventive agent. Considering that oxidative stress is a critical mediator for progression of cadmium intoxication, it is conceivable that *C.nervosum* may be beneficial to protect against oxidative renal damage caused by cadmium. The present study was set up to test this hypothesis. Firstly, fruit extract of *C. nervosum* was evaluated for phytochemical constituents and associated antioxidant properties using several *in vitro* assays. Thereafter, its potential antioxidant health benefit was examined in a rat model of cadmium-induced nephrotoxicity.

## 2. Results and Discussion

### 2.1. Phytochemical and Antioxidant Analyses of CNFE

Preliminary phytochemical screening was performed to determine groups of secondary metabolites present in CNFE. As shown in Table 1, CNFE contained a considerable amount of phenolics, moderate amounts of flavonoids, and small amount of proanthocyanidins, which were in accordance with results reported earlier [9,10,11]. The extract was subsequently tested for the association between its polyphenolic contents and antioxidant potential. Analyses using DPPH, ABTS, and FRAP in comparison with standard trolox (Table 1) demonstrated the capabilities of CNFE to act as free radical scavenger and reducing agent. These data are compatible with previous studies showing that the extracts from pulp and leaf of *C. nervosum* contain high amounts of phenolic compounds and possess antioxidant activities [8,11].

**Table 1:** Phytochemical constituents and *in vitro* antioxidant abilities of CNFE.

**Phytochemical Constituents**	**Quantity**
Total phenolics (mg GAE/g dry wt.)	61.09 ± 1.53
Total flavonoids (mg QE/g dry wt.)	32.05 ± 1.36
Total proanthocyanidins (mg CE/g dry wt.)	7.64 ± 0.33
**Antioxidant Test**	**Ability**
DPPH (mg TE/g dry wt.)	4.56 ± 0.19
ABTS (mg TE/g dry wt.)	0.28 ± 0.01
FRAP (mg TE/g dry wt.)	38.81 ± 0.85

Values are mean ± SEM of three replicates from three independent experiments. CNFE: *C. nervosum* fruit extract; GAE: gallic acid equivalents; QE: quercetin equivalents; CE: catechin equivalents; TE: trolox equivalents; dry wt.: dry weight of the extract.

### 2.2. Identification and Quantification of Phenolic Antioxidants in CNFE

In order to further characterize the major polyphenolic components of CNFE, an HPLC/DAD/MS procedure was carried out and the active compounds were identified and quantified by comparison with authentic standards. Although many natural polyphenolics exhibit antioxidant activity, the present study pays particular attention to catechin, gallic acid, and rutin because they are not just ubiquitous constituents of plants but also common components of traditional herbal remedies [12]. The antioxidant properties of these compounds including their beneficial roles on human health are also well established [13,14,15].

**Figure 1:**
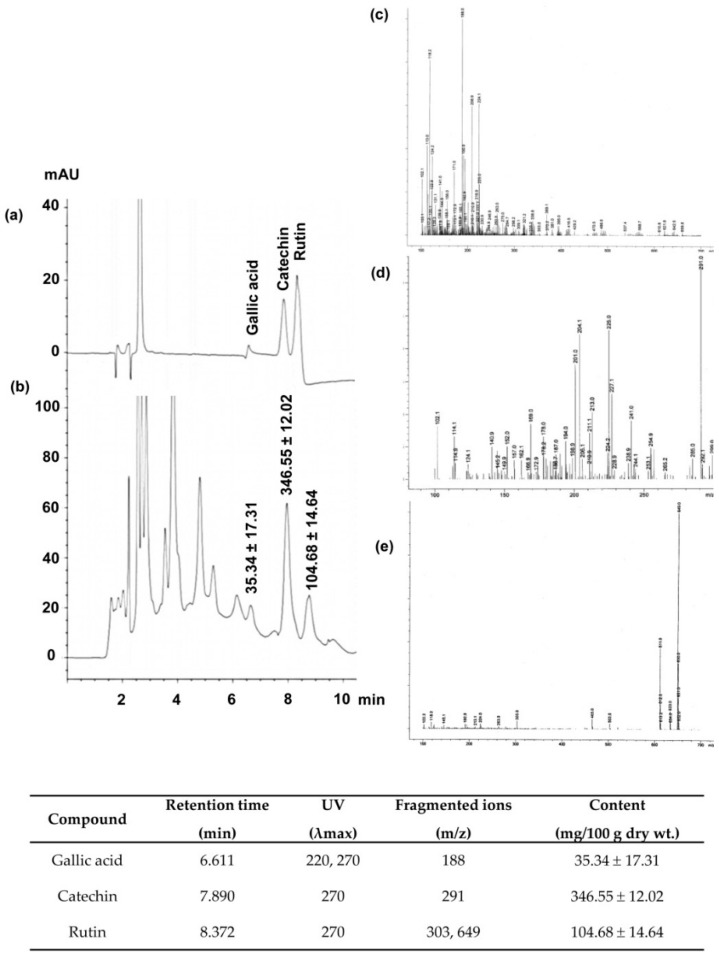
HPLC/DAD/MS analysis of *C. nervosum* fruit extract (CNFE). (**a**) Chromatogram at 270 nm of reference standards and (**b**) chromatographic peaks of the extract, (**c**–**e**) electron ionization mass spectra of gallic acid, catechin, and rutin, respectively. Numerical values in (**b**) denote the amount of individual compound detected. Data (mean ± SEM, *n* = 3) are expressed in mg/100 g dry weight of the extract. The identity, retention time, maximum UV absorption wavelength (λ_max_), and fragment ions for each identity are compiled and presented at the bottom end of the image.

Figure 1 summarizes all information obtained from HPLC/DAD/MS analysis of CNFE. The HPLC chromatogram (Figure 1a,b) shows the presence of gallic acid, catechin, and rutin at retention time 6.611, 7.890, and 8.372 min, respectively. The identity of each compound was validated as the diagnostic mass fragments obtained by LC-MS with API-ES-MS positive mode at *m*/*z* 188, 291, and 649 corresponded to the ion spectra of gallic acid, catechin, and rutin, respectively (Figure 1c–e). Quantitative analyses revealed the highest amount of catechin at 346.55 ± 12.02 mg/100 g dry wt., while those of rutin and gallic acid were 104.68 ± 14.64 and 35.34 ± 17.31 mg/100 g dry wt., respectively.

Cyanidin-3-glucoside has previously been identified as the major anthocyanin found in *C. nervosum* and has been suggested to be responsible for its antioxidant effects [9]. The present study provides additional evidence to demonstrate that *C. nervosum* is also rich in rutin and, particularly, catechin, which may be contribute to antioxidant capabilities of the extracts. To sum up, the *in vitro* data obtained herein confirm the promising role of *C. nervosum* as natural antioxidant and point out that it is useful to examine the health value of this plant in the body.

### 2.3. Effects of CNFE on General Characteristics of Cd-Intoxicated Rats

The initial body weight was very similar in all experimental groups. By the end of experiment, rats in the Cd-treated group had a percent body weight gain less (*p* < 0.05) than those in the vehicle-treated controls (Table 2). This is in line with several previous studies which reported a decrease in body weight in rats that received cadmium [16]. The present results also demonstrated that the lowered body weight gain after cadmium exposure existed despite the amount of food intake was almost identical (Table 2). This implies that the effect of cadmium is likely mediated by increasing metabolism rather than disrupting feeding mechanism. Administration of CNFE to Cd-intoxicated rats was able to protect against the unusual body weight gain, regardless of the dosages (Table 2). This protection by CNFE was also evident without modifying food consumption, indicating that CNFE is capable of counteracting the catabolic effect of cadmium and, thus, maintaining energy balance. Consistent with the body weight, the value of kidney weight recorded from Cd-treated group was significantly lower than those recorded in the vehicle and the three Cd+CNFE groups (all *p* < 0.05). No significant differences between all the groups were observed when the kidney weights to their body weights was normalized, although there was a lower tendency in the Cd-treated group (Table 2). Regarding the group that received CNFE alone, all data of body weight gain, food intake, and relative kidney weight did not show any significant differences from the vehicle group (Table 2). The findings implied that CNFE itself has no apparent influence on the baseline physical characteristics, even though it has been given continuously at 2 g/kg/day for four weeks.

**Table 2:** Effects of CNFE on general characteristics of cadmium-intoxicated rats.

Experimental Groups	Initial BW (g)	Food Intake (g/100 g/day)	BW Gain (%)	KW (g)	KW/BW (×100)
Veh	240.00 ± 3.65	4.83 ± 0.04	26.12 ± 3.77	2.26 ± 0.02	0.75 ± 0.02
CNFE	238.33 ± 3.07	4.84 ± 0.06	22.03 ± 3.02	2.29 ± 0.03	0.74 ± 0.01
Cd	240.83 ± 3.52	4.80 ± 0.08	14.15 ± 1.33 *^,†^	1.79 ± 0.02 *^,†^	0.69 ± 0.02
Cd + CNFE 0.5	241.67 ± 2.11	4.87 ± 0.04	20.14 ± 7.20	2.14 ± 0.03	0.73 ± 0.05
Cd + CNFE 1	240.83 ± 9.35	4.80 ± 0.08	22.70 ± 3.99	2.16 ± 0.08	0.73 ± 0.03
Cd + CNFE 2	243.33 ± 5.73	4.83 ± 0.06	25.81 ± 3.44	2.25 ± 0.11	0.75 ± 0.04

Values are expressed as mean ± SEM (*n* = 6). Veh: vehicle-treated control group; CNFE: *C. nervosum* fruit extract-treated group; Cd: cadmium-treated group; Cd + CNFE (0.5, 1, 2): cadmium plus CNFE-treated groups at 0.5, 1, 2 g/kg, respectively; BW: body weight. KW: kidney weight; * *p* < 0.05 *vs.* Veh, ^†^ *p* < 0.05 *vs.* Cd + CNFE groups.

### 2.4. Effects of CNFE on Renal Function and Histopathology of Cd-Intoxicated Rats

**Figure 2:**
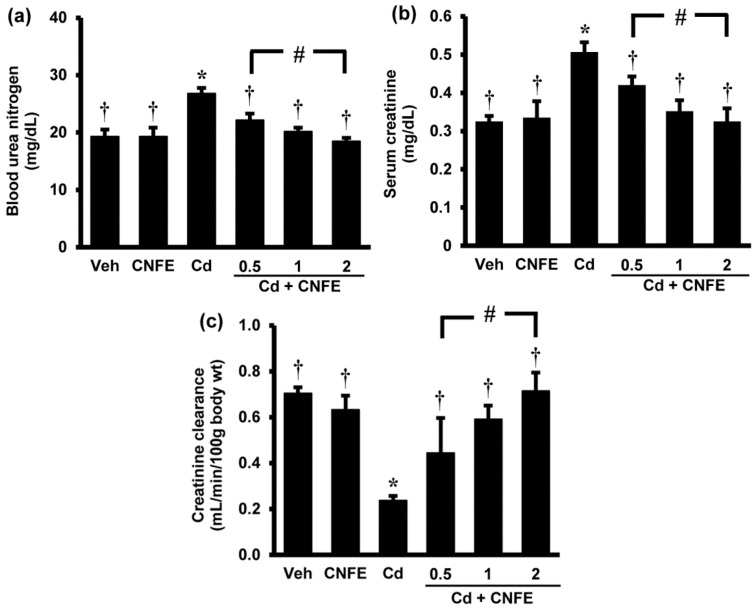
Effects of cadmium and CNFE on renal function. (**a**) Blood urea nitrogen; (**b**) serum creatinine, (**c**) creatinine clearance. Values are mean ± SEM (*n* = 6). Veh: vehicle-treated control group; CNFE: *C. nervosum* fruit extract-treated group; Cd: cadmium-treated group; Cd + CNFE (0.5, 1, 2): cadmium plus CNFE-treated groups at 0.5, 1, 2 g/kg, respectively. * *p* < 0.05 *vs.* Veh, † *p* < 0.05 *vs.* Cd, # *p* < 0.05 between Cd + CNFE.

Significant increases in the serum levels of urea and creatinine (Figure 2a,b) together with significant decrease in the creatinine clearance (Figure 2c) were observed in Cd-treated rats when compared with control rats (all *p* < 0.05). Co-administration of CNFE with cadmium significantly reduced the elevations in blood urea nitrogen and serum creatinine, and restored creatinine clearance to near normal levels when compared with Cd alone-treated rats (all *p* < 0.05). The effects of CNFE appeared to be in a dose-dependent manner; however, statistical significant difference was only reached between the lowest (0.5 g/kg) and the highest (2 g/kg) concentration of CNFE. It should also be noted here that supplementation of CNFE to normal rats for four weeks had no adverse effect on the kidney as all renal functional parameters did not show any significant differences from the baseline controls (Figure 2a–c).

**Figure 3:**
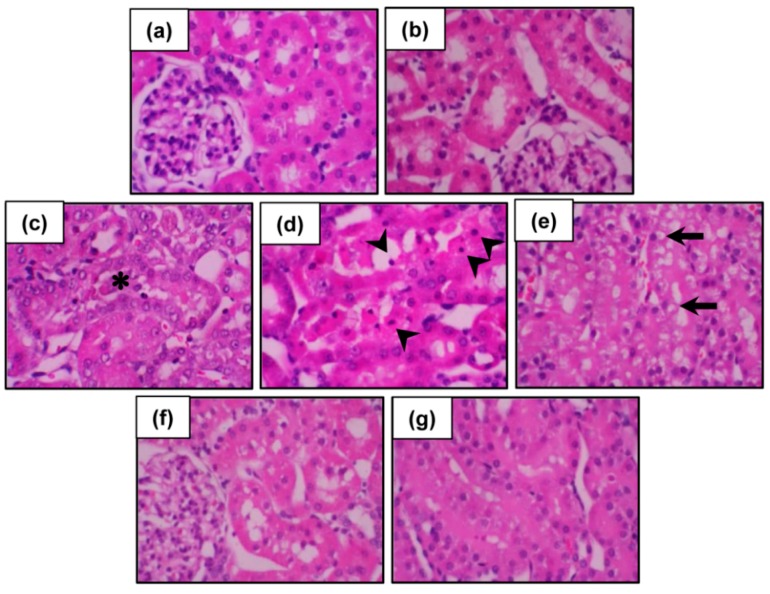
Photomicrographs of the kidneys following cadmium and CNFE treatments. Kidney tissues from (**a**) control and (**b**) CNFE-treated groups showing essentially normal tubules. In the Cd-treated group (**c**–**e**), severe tubular necrosis (asterisk), apoptosis (arrowhead), and vacuolar degeneration (arrow) were observed. Treatment with CNFE either at 1 g/kg (**f**) or 2 g/kg (**g**) markedly ameliorated all renal damages caused by cadmium. Hematoxylin and eosin (H & E), 40×.

Histopathological changes in the kidneys were further evaluated and the results are presented in Figure 3. Both vehicle-treated (Figure 3a) and CNFE alone-treated rats (Figure 3b) showed normal renal configurations. In contrast, kidney sections from Cd-intoxicated rats exhibited severe tubular necrosis, apoptosis, and vacuolar degeneration, while no definite glomerular structural changes were observed (Figure 3c–e). Treatment with CNFE at 1 g/kg (Figure 3f) and 2 g/kg (Figure 3g) markedly diminished all renal damages caused by cadmium, whereas CNFE at 0.5 g/kg showed only minimal improvement.

The results of renal function and histopathology verified the development of nephrotoxicity following cadmium exposure in the present study. The findings also suggested that cadmium decreased glomerular filtration rate, as indicated by creatinine clearance, is likely the consequence of an impaired tubular reabsorptive capacity rather than a glomerular injury. This suggestion is supported by the histological evidence of intact glomeruli but damaged renal tubules, particularly the proximal tubules. The loss of tubular integrity could promote backleak of the glomerular ultrafiltrate across the tubular epithelium, thereby decreasing the glomerular filtration rate. These results correlated well with the earlier publications demonstrating proximal tubules as the main site of cadmium deposition in the body [2,4]. Due to a large luminal area for cadmium exposing, numerous mitochondria, and a variety of transporters and receptors, the proximal tubule becomes supersensitive to the toxic effects of cadmium and proximal tubular reabsorptive dysfunction is supposed as an initial characteristic of cadmium-induced renal damage [2,4,15].

Considering that oxidative stress is a major cause of cadmium nephrotoxicity and the obtained results demonstrated the potential of CNFE to protect against renal functional and structural damages caused by cadmium, it is proposed that CNFE may accomplish the renoprotective effects mainly through its antioxidant and radical scavenging activity.

### 2.5. Effects of CNFE on Renal Oxidative Stress in Cd-Intoxicated Rats

To further confirm whether CNFE exerted its protection against cadmium nephrotoxicity via antioxidant mechanism, oxidative stress parameters in the kidney tissues were determined. As the lowest dose (0.5 g/kg) of CNFE did not seem to show significant therapeutic potency, it was omitted from this part of the study.

**Figure 4:**
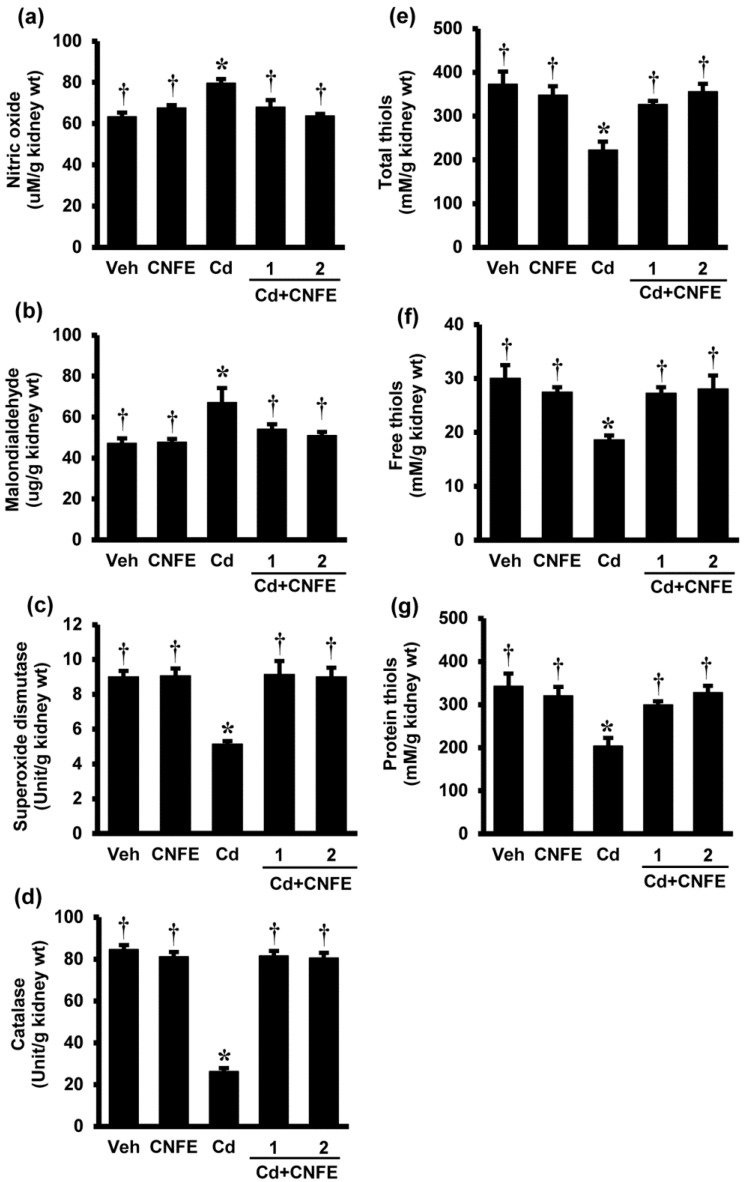
Effects of cadmium and CNFE on renal oxidative stress. (**a**) Nitric oxide; (**b**) malondialdehyde; (**c**) superoxide dismutase; (**d**) catalase; (**e**) total thiols; (**f**)free thiols; and (**g**) protein thiols. Veh: vehicle-treated control group; CNFE: *C. nervosum* fruit extract-treated group; Cd: cadmium-treated group; Cd + CNFE (1 and 2): cadmium plus CNFE (1 and 2 g/kg)-treated groups. Values are mean ± SEM (*n* = 6). * *p* < 0.05 *vs.* Veh, † *p* < 0.05 *vs.* Cd.

Figure 4 shows renal oxidative stress parameters in responses to cadmium and CNFE. The levels of NO (Figure 4a) and MDA (Figure 4b) were significantly increased in Cd-intoxicated rats compared to the vehicle controls (both *p* < 0.05). Co-treatment of CNFE with cadmium significantly decreased the rises in NO and MDA, irrespective of the doses used (all *p* < 0.05). Significant reductions in the activities of enzymatic antioxidants SOD and CAT (Figure 4c,d) including the non-enzymatic antioxidants, *i.e.*, total thiols, free thiols, and protein thiols (Figure 4e–g, respectively), were also detected in rats receiving cadmium (all *p* < 0.05). These antioxidants were significantly (all *p* < 0.05) increased and returned to the levels that were similar to their respective controls when CNFE was given along with cadmium. It is evident that the advantages of CNFE were comparable between the two doses used in this study. There were also no significant changes of all oxidative indexes in rats giving CNFE alone (Figure 4a–g).

There is now a substantial body of evidence supporting that many of the pathophysiological events triggered by cadmium are mediated through the production of reactive oxygen species (ROS) with subsequent oxidative stress [1,5,7,17]. Cadmium itself is unable to generate ROS directly because it is a non-redox reactive metal, however, it can displace endogenous metal cofactors from their active sites and these redox active metals subsequently enhance ROS formation through the Fenton and Haber Weiss reactions [1,5,7,17]. Evidence also exists that cadmium can directly cause mitochondrial dysfunction and increase ROS production [3,18]. A disturbance in antioxidant defense mechanisms has also been documented after cadmium exposure. Thiols are the major cellular antioxidant defenses and redox signaling, which is thought to be the first line of defense against cadmium toxicity [1,2,7,17]. Cadmium shows a high affinity for thiol binding and it is suggested that the depletion of intracellular thiols by cadmium is the prerequisite for ROS generation [1,2,7,17]. The decreased activities of various antioxidant enzymes such as SOD, CAT, glutathione peroxidase (GPx), and glutathione reductase (GR) have also been observed in the renal tissues of cadmium-intoxicated animals [19,20]. Cadmium-induced oxidative challenge is further supported by the observations of increased lipid peroxidation, protein oxidation, modulation of DNA, and induction of several stress response genes following cadmium exposure, which may eventually lead to cellular dysfunction and death [5,6].

Exposure to cadmium in the present study resulted in renal oxidative stress as supported by increased NO and MDA, but decreased SOD, CAT, and all forms of antioxidant thiols in renal tissues. CNFE therapy not only prevented the renal functional and structural damages caused by cadmium, but also restored all the changes in renal oxidative indexes. It is quite certain that the antioxidant potential of CNFE plays an important role in mediating its renoprotective effect. This suggestion is based on the pharmacological properties of phytochemical elements in *C. nervosum* given that previous studies, as well as our results, demonstrated the presence of nearly all active phytochemical elements of the plant such as phenolics, flavonoids, and proanthocyanidins in the extract. All these phytochemical constituents have generally been recognized to exhibit antioxidant and radical scavenging activity [21,22]. More precisely, a number of bioactive compounds identified from *C. nervosum* in this study (*i.e.*, catechin, rutin, gallic acid), as well as a previous report (*i.e.*, cyanidin-3-glycoside) [9], are also well-known for their antioxidant health benefits in several disorders associated with oxidative stress [8,13,14,15]. Further studies are still required to determine whether there are other bioactive compounds in *C. nervosum* that may contribute to the protective efficacy of the extract. The present findings, however, indicate that *C. nervosum* fruit extract confers overall protection to the kidney against oxidative damage induced by cadmium through mechanisms underlying its free radical scavenging potential integrated with preservation of the endogenous non-enzymatic antioxidant thiols including the antioxidant enzymes like SOD and CAT.

Although the present results highlight the antioxidants as the main mechanism for the benefit of CNFE, other unverified and unidentified actions of CNFE cannot be excluded. As mitochondria are the key intracellular targets for cadmium [3,18], CNFE could possibly offer protection against cadmium nephrotoxicity through a mitochondrial mechanism. Alternatively, the protective role of CNFE may be related to its effect to modify cadmium bioavailability. More specific approaches are needed to address the particular site of action and the exact mechanisms underlying the protection afforded by *C. nervosum* in this model.

It should also be mentioned here that CNFE exerted its beneficial effect at all concentrations examined in the current investigation, but the remarkable therapeutic efficacy was observed at the doses of 1 and 2 g/kg. At these high concentrations, the extract exhibited equal potential in preventing renal toxicity of cadmium. To avoid long-term exposure and harmful health effects of organic solvents, our study used water as solvent and extracted the fruits of *C. nervosum* by conventional methods. Thus, the amount of product yield obtained may not be as high as that extracted by organic solvents (e.g., ethanol) or other extraction methods (e.g., supercritical fluid extraction). We also chose to administer the extract via oral gavage, not by injection, in order to mimic real situations as much as possible. This may be the reason why we noticed the therapeutic effect of the extract at rather high concentration. However, a previous study has shown that there was no acute oral toxicity of CNFE at the dose up to 5 g/kg [9]. Our findings also provide further support to the safety profile of CNFE as both general characteristics and all biochemical parameters were unaffected even after taking CNFE at the dose of 2 g/kg for four weeks. While CNFE showed no observed adverse effects at this high therapeutic concentration, application to humans is unlikely with the current extract available because large amounts of fresh fruits need to be consumed to achieve this level. Accordingly, further studies to improve the amount of extract yield and quality including the identification and purification of functioning component (s) responsible for the beneficial effect of *C. nervosum* are necessary. In summary, the outcomes of this study demonstrate a promising health benefit of *C. nervosum* fruits and suggests that it is worth further development as a functional food and/or nutraceutical.

## 3. Materials and Methods

### 3.1. Chemicals and Reagents

Folin-Ciocalteu reagent was purchased from Merck (Darmstadt, Germany). Ferrous sulfate heptahydrate (FeSO_4_·7H_2_O), aluminum chloride hexahydrate (AlCl_3_·6H_2_O), 2,2′-azino-bis(3-ethylbenzothiazoline-6-sulphonic acid) (ABTS) were obtained from Fluka (Darmstadt, Germany). All other chemicals and reagents with analytical and HPLC grade were purchased from Sigma-Aldrich Co. (St Louis, MO, USA).

### 3.2. Plant Materials and Preparation of Cleistocalyx Nervosum Fruit Extract (CNFE)

*Cleistocalyx nervosum* were planted at Rajamangala University of Technology Lanna (Lampang, Thailand) and collected at maturation in August–September 2013. The specimen was firstly identified by Assoc. Prof. Dr. Boonsom Liawruangrath at the Faculty of Pharmacy, Chiang Mai University, in Thailand (voucher specimen no. PHAR2013CN1). The plant was further confirmed by comparing with voucher specimens of known identities (QBG 7290, QBG 17340, QBG 25139) deposited at the Queen Sirikit Botanic Garden, Chiang Mai, Thailand. The fruits were washed under running water, wiped dry, and stored at −20 °C. The pulp was subsequently isolated, weighed, lyophilized, and ground into powder. One hundred grams of the powder was extracted with distilled water for 4 h at room temperature according to previously established methods [9]. This extraction procedure was repeated thrice, and the ensuing CNFE (final yield equivalent to 8.6 g) was stored at 4 °C until analysis.

### 3.3. Phytochemical Analysis and Quantification of Polyphenolics

The CNFE was initially analyzed for its major phytochemical constituents. Total phenolic content was determined according to Folin-Ciocalteu procedure using gallic acid as standard [23]. Briefly, the extract was oxidized with Folin-Ciocalteu reagent, neutralized with 7.5% sodium carbonate, and incubated at 50 °C for 15 min. The absorbance was measured at 760 nm and phenolic content was calculated and expressed as mg of gallic acid equivalent per gram of extract in dry weight (mg GAE/g dry wt). Total flavonoid content was assessed using aluminum chloride colorimetric assay [24]. An aliquot of extract was mixed with 95% ethanol, 10% AlCl_3_·6H_2_O, and 1 M CH_3_COOK. After incubation at room temperature for 40 min, the absorbance was recorded at 415 nm. Flavonoid content was determined from quercetin standard curve and expressed as mg of quercetin equivalents per gram of extract in dry weight (mg QE/g dry wt). A modified acid/butanol assay as described by Skerget *et al.* [25] was used to quantify total proanthocyanidin content. Briefly, the assay mixture was prepared by mixing extract solution with 77 mg of FeSO_4_·7H_2_O in 500 mL of HCl:*n*-buthanol = 2:3, and incubated for 40 min at 95 °C. The absorbance was read at 540 nm after cooling and result was expressed in term of catechin equivalent (mg CE/g dry wt).

The phytochemicals of CNFE were further identified and quantified by high-performance liquid chromatography with diode array detection and mass spectrometry (HPLC-DAD/MS) on a 1100 series HPLC system equipped with MS detectors (Agilent Technologies, CA, USA). Chromatographic separation was carried out on a LiChrospher C18 column (150 × 4.6 mm; 5 µm) protected with a guard column LiChroCART^®^ (both from Merck). The mobile phase used composed of solvent A: formic acid/water (1:99; *v/v*), and solvent B: acetonitrile/water/formic acid (30:69:1; *v/v/v*). The elution profiles were set up according to the modified method of Prasain *et al.* [26]. Briefly, the initial mobile phase consisted of 100% A and was held for 5 min, a gradient was applied to 80% A in 5 min followed by a 10-min equilibration, thereafter a gradient was changed to 60% A over 40 min. The separation temperature was kept constant at 40 °C throughout the analysis, with a flow rate of 1 mL/min, and a diode array detector set to collect the signal at 270 nm. The HPLC effluent was delivered into a single quadrupole mass spectrometer via orthogonal atmospheric pressure ionization-electrospray (API-ES) interface mode at 100–700 *m/z* and step size at 0.2. The optimum electrospray ionization (ESI) conditions were as follows: ionization mode, positive (4000 V), negative (3500 V); nebulizer pressure, 60 psi; drying gas flow rate, 13 L/min; drying gas temperature, 320 °C. Polyphenolic components in the CNFE were identified and quantified by comparing the peaks UV spectra at retention time 270 nm to those of authentic standards, their structural identity were confirmed with mass spectra, and the identified compounds were quantified by comparison with peak area calibration curves of their respective standards.

### 3.4. Evaluation of Antioxidant Activities

The 2,2-diphenyl-1-picryl-hydrazyl (DPPH) radical scavenging capacity, 2,2′-azino-bis(3-ethylbenzothiazoline-6-sulphonic acid) (ABTS) radical cation decolorization, and ferric reducing antioxidant power (FRAP) assays were used to determine the antioxidant abilities of CNFE. DPPH is an antioxidant assay based on electron-transfer that produces a violet solution in ethanol. DPPH, stable at room temperature, is reduced in the presence of an antioxidant molecule, giving rise to colorless ethanol solution. The assay was carried out according to the method of Brand-Williams *et al.* [27]. The ABTS+ decolorization assay was performed according to the method described by Luximon-Ramma *et al.* [28], which based on the ability of an antioxidant to scavenge the preformed ABTS+ radicals. FRAP assay was also performed according to Luximon-Ramma *et al.* [28] based on the reduction of Fe^3+^ 2,4,6-tripyridyl-s-triazine (TPTZ) to ferrous iron (Fe^2+^) by antioxidant ability in the reducing agent. Trolox (6-hydroxy-2,5,7,8-tetramethylchroman-2-carboxylic acid) was used as a standard for all of these assay protocols. The value obtained from each assay in comparison with standard trolox, which represents antioxidant prospective of CNFE, was expressed as mg trolox equivalent/g dry weight of the extract (mgTE/g dry wt).

### 3.5. Animals and Treatments

Male Wistar rats (180–200 g) supplied by the National Laboratory Animal Center (Mahidol University, Salaya, Thailand) were kept in standard laboratory conditions, fed on a standard rodent chow, and allowed free access to water. All the animal experiments were approved by the Ethical Committee of Chiang Mai University, Thailand (project number 02/2557).

After a week of acclimatization, rats were randomly assigned into six groups (*n* = 6). Group 1 (vehicle-treated control group: Veh) was injected intraperitoneally with 0.9% normal saline solution. Group 2 (extract-treated group: CNFE) received CNFE (2 g/kg) by oral gavage. Group 3 (cadmium-treated group: Cd) was intraperitoneally injected with CdCl_2_ (2 mg/kg). Apart from CdCl_2_ injection, rats in Groups 4–6 (cadmium plus extract-treated groups: Cd+CNFE) were also supplemented orally with CNFE at the doses of 0.5, 1, and 2 g/kg, respectively. CNFE was given 1 h before CdCl_2_ injection, and all treatments were lasted for four weeks. Body weight, as well as food intake, was recorded on a daily basis for subsequent calculation of the percent body weight gain and the average amount of food consumption. On the last treatment day, rats were placed in metabolic cages for 24 h urine collections. Blood samples and kidney tissues were taken thereafter under intraperitoneal pentobarbital anesthesia.

### 3.6. Assessment of Renal Function

Blood urea nitrogen (BUN), serum creatinine, and urine creatinine were assayed using AU 480 chemistry analyzer (Beckman Coulter, Inc., Brea, CA, USA). Glomerular filtration rate (GFR) was estimated from creatinine clearance, which was calculated from the ratio of creatinine in urine/serum and the volume of urine produced.

### 3.7. Determination of Renal Oxidative Stress

The kidney tissue was homogenized in an appropriate buffer using a Potter Elvehjem homogenizer (Wheaton Science, Millville, NJ, USA). The homogenate was centrifuged at 10,000× *g* for 15 min at 4 °C, and the supernatant obtained was used for oxidative stress assays.

Nitric oxide (NO) level was quantified using Nitrate/Nitrite Colorimetric Assay Kit (BioAssay Systems, Hayward, CA, USA) according to the manufacturer’s instructions. Malondialdehyde (MDA), an index of lipid peroxidation, was estimated in the form of thiobarbituric acid reactive substances (TBARS) according to the method of Ohkawa *et al.* [29]. The non-enzymatic antioxidant thiols (both total and free thiols) were assayed directly by the method of Sedlak and Lindsay [30], while levels of protein-bound sulfhydryl groups were indirectly assessed from the difference between the values of total thiols and free thiols. The activity of superoxide dismutase (SOD) was determined using EnzyChrom™ Superoxide Dismutase Assay Kit (BioAssay Systems, Hayward, CA, USA), and catalase (CAT) enzyme activity was measured using a commercial kit from Cayman Chemical Company, USA.

### 3.8. Histopathological Examination

Kidney tissue was fixed in 10% formaldehyde buffered solution, dehydrated in ascending series of ethanol, and embedded in paraffin. Serial sections of 4 µm were cut, stained with hematoxylin and eosin (H & E), and examined under a light microscope.

### 3.9. Statistical Analysis

All biochemical assays were performed in triplicate. Results were presented as mean ± SEM. Data were analyzed by one-way ANOVA followed by Fisher’s LSD for multiple comparisons using SPSS software version 16.0 (SPSS Inc., Chicago, IL, USA). Statistical significance was assigned at a *p* value less than 0.05.

## 4. Conclusions

The present study provided support to the antioxidant abilities of *C. nervosum* and added more evidence to demonstrating catechin as one of the key components that may underlie the extract properties. Using a rat model of cadmium nephrotoxicity, the results further established a promising role for *C. nervosum* in the protection against oxidative renal damage caused by cadmium, possibly through quenching the ROS or by acting as a defense shield to protect the antioxidant defense mechanism. The study outcomes call attention to the application of *C. nervosum* as an antioxidant supplement for health preventive benefits in individuals who are at risk of cadmium contamination and may also extend to others who are encountered with oxidative stress-related disorders. The observed effects being achieved with quite high doses of CNFE, further studies on an optimization of the extract composition will also be necessary.

## Abbreviations

The following abbreviations are used in this manuscript:

CNFE: *Cleistocalyx nervosum* var. *paniala* Fruit Extract
Cd: Cadmium
GAE: Gallic Acid Equivalents
QE: Quercetin Equivalents
CE: Catechin Equivalents
TE: Trolox Equivalents
ABTS: 2,2′-azino-bis(3-ethylbenzothiazoline-6-sulphonic acid)
API-ES: Atmospheric pressure ionization-electrospray
BUN: Blood urea nitrogen
CAT: Catalase
DAD: Diode array detection
DPPH: 2,2-diphenyl-1-picryl-hydrazyl
ESI: Electrospray ionization
FRAP: Ferric reducing antioxidant power
GPx: Glutathione peroxidase
GR: Glutathione reductase
HPLC: High-performance liquid chromatography
LC-MS: Liquid chromatography mass spectrometry
MDA: Malondialdehyde
MS: Mass spectrometry
NO: Nitric oxide
ROS: Reactive oxygen species
SOD: Superoxide dismutase
TBARS: Thiobarbituric acid reactive substances
